# Posthuman Ethics for AI

**DOI:** 10.1007/s11673-025-10447-2

**Published:** 2025-08-04

**Authors:** R. Braidotti

**Affiliations:** University of Utrecht, Utrecht, The Netherlands

**Keywords:** AI, Regulation, Posthumanism, Ethics, Affirmation, Feminism

## Abstract

The impact of AI on liberal democratic social and political systems has emerged as the crucial issue of our times. So is the need to regulate general AI systems. The alliance of newly elected president Trump with the owners and CEOs of the technological sector of the U.S. economy—Elon Musk first and foremost—adds extra urgency to the issue. Digital or platform capitalism has engendered what is known as “surveillance” societies. The centralized and unchecked business model it relies on poses serious existential threats to our collective futures. There is widespread consensus today, not only in academia but also in progressive social circles, that we need both traditional and algorithmic sources of resistance to monopolies and the centralization of technological powers. We also need more experimentation with alternative ways of developing and governing the AI dimension of our lives.

The impact of AI on liberal democratic social and political systems has emerged as the crucial issue of our times. So is the need to regulate general AI systems. The alliance of newly elected president Trump with the owners and CEOs of the technological sector of the USA economy—Elon Musk first and foremost—adds extra urgency to the issue. Digital or platform capitalism has engendered what is known as “surveillance” societies. The centralized and unchecked business model it relies on poses serious existential threats to our collective futures. There is widespread consensus today, not only in academia, but also in progressive social circles, that we need both traditional and algorithmic sources of resistance to monopolies and the centralization of technological powers. We also need more experimentation with alternative ways of developing and governing the AI dimension of our lives.

I am writing this from an assumption of *technophilia*, as I share pride and joy in the achievements of our science and technologies. Yet, I also write out of concern for the issues of governance and control engendered by this particular form of capitalism. In this short essay I argue for the need of an ethics adequate to the complexities of our times and a political system of democratic accountability for the techno-universe we have engineered. From a posthuman perspective, I call this affirmative ethics as a communal praxis. But let me first spell out the difference between transhumanism and posthumanism.

## The Transhumanist Delirium

Contemporary technologies in advanced capitalism spell the end of the modernist interaction between humans and the machines they produce. In high modernity, the technological other was distinct from the human self: it is represented and dichotomized as the other of the human—threatening but also fatally attractive. But a new social imaginary surrounds the digital universe. Contemporary technology is invasive in an immersive, all-pervasive manner that blurs all distinction between selves and technological extensions. Digital technologies take over and boost basic human faculties, neural and perceptive abilities, computational and visualization functions.

The transhumanist hubris at work in these technologies aims to redefine, enhance, or replace the human, multiplying it over a thousand plateaus of possible expansion. As a consequence, practices of human enhancement are now quite common, for instance in medicine, healthcare and beauty care but also in AI and industrial applications. By improving and perfecting the human in advanced capitalism, transhumanists claim they accomplish the Enlightenment project of humans evolving through human brain–AI interaction. As a result we cannot really mark a dialectical demarcation between the human and the technological other: our interaction has become far more “rhizomatic.” This all-pervasive relation to humans makes AI technological systems far more complicated to deal with than previous technologies. Moreover, there is a tendency to want to leave planet Earth, by investing billions of dollars in realizing the fantasy of establishing human settlements on other planets, starting from the Moon and taking it all the way to Mars.

In my view there are several things wrong with the transhumanist project. First, it is strongly utopian by aiming at the long term (“long termism”), neglecting the present predicament of environmental deterioration and depletion of sources on Earth. Secondly, it ignores any social and economic issues linked to the impact of technology.

On the contrary, a *posthuman* critical approach focuses on the present while it does not isolate the digital and technological from its grounding in the environmental dimension. Nor does it ignore the social-economic implications of these technologies. I refer to this complex interaction as the posthuman *convergence* of the digital, technological, environmental, and social. I call for an adequate ethical and political critique to come to terms with the posthuman convergence. In other words, a posthuman critical approach puts a strong emphasis on ethical values and on regulation and governance.

My roots in 1990’s cyberfeminism have made me aware of the enormous disruptive as well as the revolutionary potential of contemporary technologies. I argue that one of the points of resistance consists in exploring and exploiting precisely the *liberatory* potential of these immersive technologies. The reason being that the enhancements and expansions of the human do not happen in a vacuum. They happen in a world where power relations are structured by sexualized, racialized, and naturalized hierarchies and by an extraordinary concentration of capital and profits.

There is an inherent contradiction built in the transhumanist project, between on the one hand the delirious promises of human enhancement and expansion into outer space and on the other hand the traditional and even reactionary power formations that support them. The term “techno-feudalism” has been evoked to describe the existential threats posed by the transhumanist project of the tech billionaires.

This tension between the liberation of new potentials and the entrapment of new forms of authoritarian governance is, in my assessment, a defining feature of the posthuman convergence. We live amidst incredible paradoxes, which we as feminist, anti-racist, and democratic-minded critical thinkers need to analyse very lucidly. It would be naïve to be seduced or coerced into a new economic and social order that reiterates the worst features of centralized power and of the old extraction economy by transposing it to other planets (if such a project is even possible). As critical thinkers we need to resist a new kind of extra-terrestrial oligarchic structure of command, ordered around the delirious longings, and the acute fears, of the old patriarchs. The right-wing politicians and the tech billionaires seem to be haunted by deep anxieties about female empowerment, ethnic replacement, and the end of the Western world. Yet, it is both possible and necessary to come up with more inspirational and progressive scripts for our collective futures.

## New Ethical and Political Scripts: Affirmation and Diversity

I am all in favour of posthuman transformation. But, there is a but. The posthuman predicament requires participation, inclusion, consultation, and regulation.

The most problematic aspect of the promise of human “liberation” that is driving the current expansion of AI, is that it surreptitiously introduces a unified notion of the “human.” This AI-driven corporate pan-humanism, in fact wipes out crucial differences in the politics of location of the humans involved. This means it cuts their access to power and technological privileges and hence in their abilities to play a role in redefining the kind of humans we are in the process of becoming.

This spurious reinvention of pan-humanity is problematic because it supports neoliberal economic agendas and abandons the cause of including minority and marginalized groups. Artificial intelligence programmes attempt to eradicate “difference” in the name of a vulnerable and insurgent “humanity,” which happens to coincide with the interest of capitalist market economies. My response is to raise the question: how can AI work more affirmatively by accepting human diversity? And what kind of techno-imaginary do we need to achieve that aim?

The political culture of the left, of which feminism and anti-racism are major components, has a difficult relationship to technologies and to AI. Automation and robotics are conventionally seen as potentially hostile to human labour and as especially threatening for the devalorized, marginalized, and under-paid “others,” the sexualized and racialized naturalized others: women, LBGTQ+ people, indigenous, Black, and colonized people. Advanced technologies are often perceived as an instrument that can potentially make their fate worse. The issue here is that the “human” both threatened and enhanced by AI-driven technologies is not a single, neutral, or undifferentiated term but an internally fractured one. “We” are not all human in the same way, to the same extent, or with the same effects: there are enormous differences of power and privilege in terms of how “we” inhabit our common humanity. A great deal of humans—especially the “others” mentioned above—are de-humanized or relegated to a category of being “not fully” human. Consequently, they can only confront the new smart technologies with an extra degree of trepidation.

In order to diversify the notion of a pan-humanity united by and in fear of AI-driven smart technologies, and to ensure more accountability and inclusion, we need to reflect more critically about the becoming-posthuman of sectors of the population who were never recognized as fully human to begin with.

For instance, as a member of the LBGTQ+ community, and as a feminist committed to social justice and transformative politics, I am quite worried about the criteria of selection of the social categories and single individuals who get to be enhanced. Indeed, who get to be improved, through which technological procedures, and for what ultimate purpose? A generalized appeal to “the defence of humanity” does not cut it for me. My concern is that many people who are not considered fully human are and will remain excluded from this new pan-humanity bonded to AI.

AI is a system of computational statistics to the nth power. Recent scholarship has shown that the algorithms underpinning AI technologies are programmed to favour outcomes that align with dominant cultural norms, erasing or marginalizing difference as “error” or “noise” that disrupts the code itself as it diverges from the “dominant” data set. This is where the exclusionary, discriminatory, and neo-colonial logic of AI becomes most apparent: in its capacity to classify, sort, and hierarchize human populations. Reiterating the worst aspects of the Eurocentric mindset, AI emerges as a tool for the continuation of masculinist, colonial, imperialist agendas. Marginalized groups—be they the socially dis-enfranchised and economically excluded, the racialized and migrant minorities, women and LGBTQ+ communities, or disabled and neurodivergent individuals—are at particular risk of being excluded from the so-called “universal” datasets that inform the humanity designed by AI development.

Another worrying issue is the necro-political aspect of this recreation of humanity, for instance in the use of AI in surveillance and the automatization of border control. And also in military drone strategies and automated weapons in determining which subject is “suspect.” In all these cases, the use of a dominant data set becomes problematic as it uses AI to calculate the statistical probability of certain “characteristics” of the categories given as suspect. The racialized bias of these face-recognition algorithms and other AI-fed devices of detection, has become a source of major concern.

My point of reference to redefine humanity within diversity is to emphasize the embodied, embedded, relational, and affective structure of all living entities, humans included. The older debate between those who are “proud to be flesh” and those who “want to be wired” has become polarized through the transversal force of AI technologies. To be happy with just being anthropomorphically human is simply out of the question for big tech transhumanists. They prefer to enhance, redesign, and replace—the faster and sooner, the better.

And yet, this is the point that I want to emphasize time and again: we are never “just human.” All of us are embodied and embedded, humans and non-humans alike. All living entities are always embedded and intermingled with nonhumans, whether organic—bacteria, animals, and plants. This even extends to technological non-humans like algorithms, codes, and AI driven platform and interfaces. We need to think a lot more systematically about what contemporary embodied and embedded, relational and affective subjects are capable of becoming, as heterogeneous assemblages that involve high degree of technological mediation.

## Affirmative Ethics

The ethics of affirmative posthuman thought is simple: “we” may well be in this predicament together, but “we” are not one and the same. We differ within this category of the human, as we always have. The transhumanist return to a contemporary version of eighteenth century ideas about the human as a new universal fails three times over: firstly in terms of disrespecting the diversity of humans; secondly by neglecting the complexities of contemporary AI technologies; and thirdly by ignoring its impact on the environment.

I recommend that we build on the remarkable archive of critical theories, new materialism, critical posthuman thought, and non-deterministic philosophies of life, to deal with the challenges of AI-driven humanity. Instead of a corporate vision of a fragile pan-humanity, we need a more diverse and creative idea of what we are capable of becoming. A posthuman ethics of becoming is both generative and affirmative, while remaining critical.

Becoming more affirmative means that, instead of imposing one single model of an AI-enhanced pan-humanity, we analyse and regulate the apparatus that is being developed. Alternative algorithmic systems and AI models have to be established, not on the basis of a neo-universal corporate idea of pan-humanity assisted by AI, but as an intricate web of heterogeneous human and non-human relations.

The challenge consists in reimagining AI as an agent of radical relationality, which may allow marginalized groups to reclaim it as a tool for their own empowerment. Artificial intelligence need not be entrapped within neoliberal functionalism; it can be regulated, for instance along the lines proposed by the European Commission. It need not function as an apparatus of control and profit but can become an instrument of affirmation and diversity.

The cyberfeminist in me knows that the possibility of resistance lies in reappropriating the algorithmic logic itself and play it against the perennial patriarchal fantasy of instilling one dominant code. Since the 1990’s there has been a movement of artists, hackers, activists, and technologists working to rupture, disrupt, and subvert the big capitalist mainframe use of AI from within. The time has come to create alternative infrastructures that prioritize diversity, social inequalities, minority voices, open access, decentralized networks, and community-driven projects.

Affirmative ethics is the posthuman philosophy of the big “yes” to multiple ways of transforming the human.

## Data Availability

NON APPLICABLE

